# Minimally invasive closure of transthoracic ventricular septal defect: postoperative complications and risk factors

**DOI:** 10.1186/s13019-021-01415-z

**Published:** 2021-03-19

**Authors:** Chunnian Ren, Chun Wu, Zhengxia Pan, Yonggang Li

**Affiliations:** 1 Department of Cardiothoracic Surgery, Children’s Hospital of Chongqing Medical University, Chongqing, 400014 No.136, Zhongshan 2nd Road, Yuzhong Dis P.R. China; 2 Ministry of Education Key Laboratory of Child Development and Disorders; National Clinical Research Center for Child Health and Disorders (Chongqing); China International Science and Technology Cooperation base of Child Development and Critical Disorders, Chongqing, P.R. China; 3 Chongqing Key Laboratory of Pediatrics, Chongqing Medical University, Chongqing, P. R. China

**Keywords:** Congenital heart diseases, Ventricular septal defect, Risk factors, Surgery, Cardiac intervention

## Abstract

**Objectives:**

To summarize and analyze the clinical characteristics of postoperative complications after minimally invasive closure of transthoracic ventricular septal defect, and to explore the risk factors for its occurrence.

**Methods:**

Retrospectively analyzed the clinical data of 209 patients underwent transthoracic ventricular septal defect closure performed in the Department of Cardiothoracic Surgery, Children’s Hospital of Chongqing Medical University from January 2018 to January 2020, obtained relevant clinical data from the electronic medical record system and summarized their postoperative complications. And used univariate logistics regression and multivariate logistics regression to analyze the risk factors of its occurrence.

**Results:**

The postoperative hospital stay of 27 patients was longer than 9 days. Residual shunt occurred in 33 patients recently after operation. One patient underwent surgical treatment again because of mechanical hemolysis after the operation. Two patients were re-operated 1 month and 10 months after surgery because of persistent moderate to severe aortic regurgitation. After surgery, 3 patients underwent pericardiocentesis due to a large amount of pericardial effusion, and 2 patients developed a new atrioventricular block after the operation. No other serious adverse events occurred. Multivariate logistic regression analysis showed that the size of VSD defect (OR: 1.494, 95% Cl: 1.108–2.013, *P* value: 0.008) was related to long postoperative hospitalization. The residual shunt is related to the size of the occluder (OR: 1.452, 95%Cl: 1.164–1.810, *P* value: 0.001). In the univariate logistics regression analysis, no risk factors related to serious adverse events were found.

**Conclusions:**

The minimally invasive closure of transthoracic ventricular septal defect is very effective, with no mortality and low incidence of serious adverse events after surgery. The size of the defect is related to the long postoperative hospitalization, and the size of the occluder is related to the residual shunt in the early postoperative period. No risk factors related to the occurrence of serious adverse events after the operation were found.

## Introduction

Ventricular septal defect (VSD) is one of the most common congenital heart diseases, accounting for about 20% of congenital heart disease [1]. The treatment methods of ventricular septal defect include repair of ventricular septal defect under cardiopulmonary bypass, minimally invasive closure of transthoracic ventricular septal defect and interventional closure of ventricular septal defect by percutaneous puncture. In recent years, minimally invasive closure of transthoracic ventricular septal defect has been widely used in China. This procedure is suitable for almost all patients. It does not require cardiopulmonary bypass, small surgical incisions, quick postoperative recovery, no X-ray exposure and wider indications than interventional closure, which has been increasingly recognized by clinicians and patients/parents [2, 3]. Chen’s research of 1090 patients undergoing this procedure is the largest report [4]. However, no studies specifically summarized the postoperative complications and corresponding risk factors of this operation. The purpose of this study was to evaluate the complications of minimally invasive closure of transthoracic ventricular septal defect in a short-term single-center cohort and to determine the corresponding risk factors.

## Methods

### Source of data and participants

Retrospectively analyzed the clinical data all patients underwent transthoracic ventricular septal defect closure performed in the Department of Cardiothoracic Surgery, Children’s Hospital of Chongqing Medical University from January 2018 to January 2020. The study was conducted in accordance with the Declaration of Helsinki and was approved by the Ethical Committee of Chongqing Medical University. Written informed consent was obtained from the patients’ parents before inclusion in this study. The research data were anonymized, and personal identifiers were completely removed. Patients were selected according to the following indications: All patients were diagnosed by transthoracic echocardiography before surgery, VSD size was 3-8 mm, clear left to right shunt and with or without mild to moderate pulmonary hypertension. Exclusion criteria: ①The size of the VSD is greater than 8 mm or less than 3 mm; ②The age is less than 5 months; ③Combined with other types of complicated congenital ④Patients who refuse to undergo this operation.

We included a total of 209 patients. We collected the patient’s information from the hospital’s electronic medical record system, ① basic information: gender, age, weight ②preoperative data: defect size, defect type, with or without pulmonary hypertension and with or without combined with aortic valve prolapse ③intraoperative data: the size of the defect, the size of the occluder, the type of the occluder, other operations during the operation, the surgeons and the operation time ④postoperative data: residual shunt, postoperative hospital stay, postoperative serious adverse events ⑤follow-up data.

The types of VSD defects can be divided into three groups: perimembrous defect, subarterial defect and muscular defect. The sonographer will initially assess whether the child has pulmonary hypertension before surgery. Pulmonary artery systolic pressure (PASP) > 25 mmHg is considered to have pulmonary hypertension. Postoperative outcomes and complications were evaluated according to international standards [5], including postoperative hospital stay, residual shunt and serious adverse events. For severe residual shunt, reoperation, reintubation, wound infection, post-pericardiotomy syndrome, complete atrioventricular block, unplanned second surgery, heart block requiring permanent pacemaker, circulation failure and death are considered serious adverse events.

Transthoracic cardiac color Doppler ultrasound and electrocardiogram were routinely reviewed 3–7 days after surgery, and cardiac color Doppler ultrasound and electrocardiogram were followed up in the outpatient clinic at 1, 3, 6, and 12 months after surgery. All children received oral aspirin 3–5 mg/kg daily for 3–6 months.

### Surgical approach

After anesthetized the child in the operating room, took the supine position, and evaluated the position, size, upper and lower edges of the defect and the aortic valve by TTE. A small incision of 2 to 4 cm in the middle of the skin (the skin incision of the perimembrous VSD was located in front of the lower end of the sternum and entered the mediastinum through the lower part of the sternum. The skin incision of the subarterial VSD was located in front of the middle sternum, and the skin incision was pulled to the left to enter the mediastinum through the third intercostal space on the left edge of the sternum.) Cut and suspend the pericardium. After 0.5 mg/kg heparinization, TEE guided the positioning of the right ventricular surface purse-string suture, selected domestic eccentric or equilateral occluder (Shanghai Shape Memory Alloy Co, Ltd), trocar punctured the right ventricular wall, removed the needle core, and under TEE monitoring, guided the wire from the right ventricle through the ventricular septal defect to the left ventricular cavity, placed the sheath, released the left ventricular surface umbrella disk, gently pulled back to make the left umbrella disk close to the ventricular septum, retracted the sheath to the right ventricular cavity, released the occluder waist and right ventricular surface umbrella tray. The “Mark” of the eccentric umbrella disc is far away from the aortic valve annulus. Subatrial ventricular septal defect or ventricular septal defect edge less than 2 mm from the aortic valve annulus choosed eccentric occluder, and the rest choosed equilateral occluder. For tunnel-type ventricular septal defects with inconsistent diameters of the left and right ventricular surfaces, the occluder is selected based on the right ventricular surface. TEE immediately evaluated the therapeutic effect, including residual shunt, function of the surrounding structure of the occluder, hemodynamics, stability of the occluder, pericardial effusion, heart rhythm, etc. If there is residual shunt, increase the occluder model. If it meets the quality control standards: no residual shunt, mild to moderate valve regurgitation, stable occluder, no complete atrioventricular block, no pericardial effusion, removed the occluder from the delivery device, and half protamine neutralized heparin. The surface of the right ventricle is ligated with a purse-string suture, stop bleeding and closed the chest. If it does not meet the quality control standards, transfer to surgery under cardiopulmonary bypass.

### Statistical analysis

The Kolmogorov-Smirnov test is used to verify whether our data were normally distributed or not. For normally distributed data, we used the means and standard deviations to describe variables. For not normally distributed data, we used medians and ranges to describe variables. Frequencies and percentages were used for categorical data. The related risk factors of surgical complications were analyzed by univariate logistics regression. In order to avoid meaningful factors from being screened out, we set *P* < 0.1 as statistically significant. The statistically significant factors of univariate analysis were included in the multivariate logistics regression analysis, and the forward LR method is used. In the end, the statistically significant factors were independent risk factors. *P* < 0.05 means the difference is statistically significant. The statistical analyses were performed using SPSS version 24 (IBM, Armonk, NY, U.S.A).

## Results

### Patient characteristics

A total of 209 patients received minimally invasive closure of transthoracic ventricular septal defect (Table 1). 55% were men, the median age of surgery was 29 months, the median weight was 12 kg, preoperative transthoracic ultrasound revealed 76.6% of the defects were perimembrous defects, and only 2.4% of muscular defects. Before surgery, 24.4% of patients had mild to moderate pulmonary hypertension. 39.2% of patients suffered from aortic valve prolapse, and 80.5% of them had mild aortic regurgitation.

**Table 1:** Patient characteristics

	All patients (***n*** = 209)
**Gender**	
**Male**	**115 (55%)**
**Female**	**94 (45%)**
**Weight (kg)**	**12 (10.00–14.00)**
**Age (month)**	**29 (15.00–42.50)**
**Type of defect**	
**Perimembrous defect**	**160 (76.6%)**
**Subarterial defect**	**44 (21.1%)**
**Muscular defect**	**5 (2.4%)**
**Aortic valve prolapse**	
**Yes**	**82 (39.2%)**
**No**	**127 (60.8%)**
**Mild aortic regurgitation**	
**Yes**	**79 (37.8)**
**No**	**130 (62.2)**
**Pulmonary hypertension**	
**Yes**	**51 (24.4%)**
**No**	**158 (75.6%)**

### Operative characteristics

A total of 7 surgeons were included in the study. 11% of patients underwent transthoracic atrial septal defect closure during the operation, 3% underwent arterial duct ligation during the operation, and 1 patient with diaphragmatic bulge underwent thoracoscopic surgery at the same time. The median size of the VSD defect was 5 mm, and the median size of the occluder used during the operation was 6 mm. Three patients used 2 occluders during the operation due to the presence of two defects, and 30.1% of the patients used eccentric occluders (Table 2).

**Table 2:** Operative characteristics

	All patients (n = 209)
**Combined other operations**	
**VSD**	**180 (86.1%)**
**ASD**	**23 (11%)**
**PDA**	**3 (1.4%)**
**ASD and PDA**	**2 (1.0%)**
**Diaphragm swelling**	**1 (0.5%)**
**Defect size (mm)**	**5 (4.00–6.00)**
**Occluder size (mm)**	**6 (5.00–7.00)**
**Type of occluder**	
**eccentric**	**63 (30.1%)**
**Equilateral**	**146 (69.9%)**
**Surgeons**	
**1**	**22 (10.5%)**
**2**	**13 (6.2%)**
**3**	**53 (25.4%)**
**4**	**56 (26.8%)**
**5**	**22 (10.5%)**
**6**	**34 (16.3%)**
**7**	**9 (4.3%)**
**Operation time (min)**	**60 (50–80)**
**Postoperative hospital stay (day)**	**7.88 ± 2.93**

### Complications

After surgery, one patient was intubated for 95 h due to severe lung infection and supraventricular tachycardia, and another patient was intubated for 406 h due to severe pulmonary hypertension and severe lung infection. The average postoperative hospital stay was 7.88 ± 2.93 days, which was related to our hospital’s clinical path and postoperative health management. For those children whose hospital stay was longer than 9 days, these patients had complications after surgery, which we defined as postoperative Long hospital stay. A total of 27 patients were hospitalized for more than 9 days after surgery. The main reason for the prolonged hospital stay was infection (respiratory tract). A total of 33 patients had residual shunt after operation, and the residual shunt width was 1 to 2.3 mm, flow velocity 1.83 to 3.46 m/s. After 1–12 months of follow-up, except for 4 patients who were lost to follow-up, 2 patients persisted with residual shunt, but all had a small residual shunt < 2 mm, and the residual shunt of the remaining patients closed spontaneously. One patient had mechanical hemolysis after surgery and immediately underwent a second operation to remove the occluder. Two patients were re-operated 1 month and 10 months after surgery because of persistent moderate to severe aortic regurgitation. A large amount of pericardial effusion was found in 3 patients after the operation and received pericardiocentesis, and 2 patients developed a new atrioventricular block after the operation. And no patients died. Serious adverse events occurred in 3.3% of patients after surgery and follow-up.

### Predictors

Univariate analysis regression analysis was performed to determine the risk factors of long postoperative hospitalization, residual shunt and serious adverse events after surgery, and the meaningful factors of univariate analysis regression analysis were incorporated into the multivariate analysis regression analysis. Long postoperative hospitalization after surgery is defined as the length of hospital stay greater than 9 days. Due to the rarity of serious adverse events, we only conduct univariate analysis regression analysis on it. The analysis results of long postoperative hospitalization, residual shunt and serious adverse events are shown in Table 3. The size of VSD defect (OR: 1.494, 95% Cl: 1.108–2.013, *P* value: 0.008) is related to long postoperative hospitalization after surgery. The postoperative residual shunt is related to the size of the occluder (OR: 1.452, 95% Cl: 1.164–1.810, *P* value: 0.001). In univariate analysis, no risk factors related to serious adverse events were found.

**Table 3:** Univariate and multivariate logistic regression for the postoperative complications

Long-term hospitalization					residual shunt				serious adverse events	
	Univariate analysis		Multivariate analysis		Univariate analysis		Multivariate analysis		Univariate analysis	
	**OR(95%CI)**	***P*** **Value**	**OR(95%CI)**	***P*** **Value**	**OR(95%CI)**	***P*** **Value**	**OR(95%CI)**	***P*** **Value**	**OR(95%CI)**	***P*** **Value**
**gender**	**0.571 (0.244–1.337)**	**0.197**	**NA**		**1.126 (0.603–2.103)**	**0.71**	**NA**		**1.659 (0.362–7.605)**	**0.514**
**age**	**0.972 (0.947–0.998)**	**0.032**	**NA**		**1.000 (0.987–1.013)**	**0.979**	**NA**		**1.005 (0.979–1.033)**	**0.706**
**weight**	**0.936 (0.847–1.035)**	**0.199**	**NA**		**0.986 (0.929–1.046)**	**0.63**	**NA**		**0.969 (0.822–1.141)**	**0.703**
**pulmonary hypertension**	**1.667 (0.697–3.983)**	**0.251**	**NA**		**1.495 (0.745–3.001)**	**0.258**	**NA**		**4.397 (0.950–20.350)**	**0.058**
**Aortic valve prolapse**	**0.747 (0.318–1.752)**	**0.502**	**NA**		**0.590 (0.303–1.149)**	**0.121**	**NA**		**0.610 (0.116–3.221)**	**0.56**
**Type of defect**	**1.387 (0.651–2.952)**	**0.396**	**NA**		**0.650 (0.319–1.322)**	**0.234**	**NA**		**1.120 (0.256–4.895)**	**0.881**
**Combined other operations**	**1.668 (0.944–2.947)**	**0.078**	**NA**		**1.095 (0.632–1.898)**	**0.746**	**NA**		**1.654 (0.694–3.939)**	**0.256**
**Defect size**	**1.532 (1.143–2.054)**	**0.004**	**1.494 (1.108–2.013)**	**0.008**	**1.456 (1.154–1.838)**	**0.002**	**NA**		**1.495 (0.889–2.514)**	**0.13**
**Occluder size**	**1.337 (1.017–1.758)**	**0.038**	**NA**		**1.452 (1.164–1.810)**	**0.001**	**1.452 (1.164–1.810)**	**0.001**	**1.526 (0.919–2.537)**	**0.103**
**Type of occluder**	**0.788 (0.315–1.969)**	**0.609**	**NA**		**0.689 (0.339–1.402)**	**0.304**	**NA**		**0.376 (0.044–3.192)**	**0.37**
**Operation time**	**1.004 (0.988–1.021)**	**0.607**	**NA**		**1.016 (1.004–1.029)**	**0.012**	**NA**		**1.022 (0.998–1.047)**	**0.068**
**Surgeons**	**0.994 (0.772–1.278)**	**0.961**	**NA**		**1.127 (0.926–1.371)**	**0.232**	**NA**		**1.520 (0.915–2.525)**	**0.106**

## Discussion

At present, existing studies have summarized the minimally invasive closure of transthoracic ventricular septal defect. Xu and colleagues [6] have reported a series of 235 cases of minimally invasive closure of transthoracic ventricular septal defect of children with a success rate of 94.9%, Chen and colleagues [4] reported 1090 cases of minimally invasive closure of transthoracic ventricular septal defect with a success rate of 94.8%. They all concluded that the method is safe and reliable. Our research further confirmed their conclusions. However, there is no research to thoroughly explore the postoperative complications of this operation and the corresponding risk factors. This study further summarizes and analyzes the postoperative complications of patients with minimally invasive closure of transthoracic ventricular septal defect. Postoperative complications include long-term hospitalization after operation, residual shunt and serious adverse events, the corresponding risk factors were analyzed.

Subarterial VSD is close to the aortic valve. The defect can cause partial loss of support of the aortic valve, prone to aortic valve prolapse, which may cause aortic valve insufficiency. After the occluder is placed, the two umbrella discs are easy to clamp the aortic valve, which affects the function of the valve and worsens the regurgitation. Therefore, preoperative transesophageal ultrasound needs to carefully evaluate the distance between the defect edge and the aortic valve and whether there is aortic valve prolapse and regurgitation flow. In this study, all 44 cases of subarterial VSD were successfully sealed, which is related to the preoperative transesophageal ultrasound screening to exclude cases of obvious aortic valve prolapse.

cAVB is one of the serious complications. There are early literature reports that the incidence of this complication accounts for 1 to 5% of patients undergoing interventional closure of ventricular septal defect [7–9]. Chen’s study shows the incidence of this complication accounts for 1.6% of patients undergoing minimally invasive closure of transthoracic ventricular septal defect [4], our research shows it is 0.9%. According to the report of Zhou [10], the possible cause of cAVB is the result of mechanical damage caused by the catheter or the occluder itself. Compared with interventional closure of ventricular septal defect, minimally invasive closure of transthoracic ventricular septal defect has a shorter transmission path, which affects the conduction system. The potential risk of mechanical damage to the conduction system is smaller, so the incidence of cAVB will be lower. Aortic regurgitation is another serious complication. Fang [1] reported that no moderate to severe aortic regurgitation was found after traditional surgical repair. Our study showed that 2 patients had moderate to severe aortic regurgitation during the postoperative and follow-up period and were treated again by surgery. Considering that the lower edge of the aorta of VSD is short and the distance between the occluder and the aortic valve is close, the eccentric surface of the occluder is adjusted to the apex during the operation to avoid the occurrence of aortic regurgitation in the VSD near the aortic valve.

As one of the important indicators to evaluate the effect of surgical treatment of ventricular septal defect, residual shunt has received extensive clinical attention. In this study, 15.8% of patients were found to have residual shunts after surgery. All patients were followed up for 1 year. Except for 4 patients who were lost to follow-up, 2 patients had residual shunts, and the remaining shunts were closed spontaneously. Deng [11] reported that the incidence of residual shunt after repair of ventricular septal defect under cardiopulmonary bypass was 31.2%, Dodge-khatami [12] reported 35%; Mahimarangaiah [13] reported the incidence of residual shunt after interventional closure of ventricular septal defect by percutaneous puncture was 29.4%, and Bai [14] reported that it was 6.8%. Our results are similar to previous studies, and all the residual shunts found are low-to-moderate, and there is no need for additional surgical intervention after surgery and follow-up.

Long-term hospitalization, residual shunt, and serious adverse events are considered to be important indicators of postoperative complications [5]. For these indicators, we analyzed their corresponding risk factors. In our study, serious adverse events occurred in 3.3% of patients, including 1 patient who had mechanical hemolysis after surgery and 2 patients who underwent surgery for moderate to severe aortic regurgitation. Two patients who had cAVB after operation, 3 patients who underwent pericardiocentesis due to moderate to severe pericardial effusion after operation. Because of the rarity of serious adverse events in our study, we only performed a univariate logistics regression. Previous studies believed that low body weight was a risk factor of serious adverse events and prolonged hospital stay after traditional surgical repair [15]. However, our results showed that no risk factors related to transthoracic closure were found, so we believe that for transthoracic closure, patients with low body weight are not at greater risk of serious adverse events after surgery. This study shows that the risk factors of early residual shunt after minimally invasive closure of transthoracic ventricular septal defect are related to the size of the occluder. The specific reason is not clear and needs to be further studied in the future. Long postoperative hospitalization is related to the size of the VSD defect. Long-term hospitalization after surgery is defined as > 9 days. Within the provisions of the clinical pathway, our hospital’s experience is that the patient will continue to be hospitalized for observation for about 1 week after the operation. If the patient has no surgery-related complications, respiratory infections and heart rhythm, etc. Can be discharged from the hospital. The reason for this is to make the families of the patients more at ease. The main reason for the prolonged postoperative hospital stay is respiratory tract infection. We believe that the larger the VSD defect, the greater the left to right shunt flow in children and the worse the cardiopulmonary function, the greater the difference in hemodynamics after the operation, the immune function of the body will be further reduced, and the postoperative hospital stay will be further prolonged.

At present, for minimally invasive closure of transthoracic ventricular septal defect, the largest report we have known is Chen’s analysis of the efficacy of 1090 patients, but there is no research specifically to discuss and summarize the postoperative complications and risk factors of this operation. We evaluated the complications of the operation based on the three indicators of postoperative hospital stay, residual shunt and serious adverse events and analyzed their risk factors separately. However, due to the rarity of serious adverse events, we only performed a univariate logistics regression on them. In future research In order to truly determine its risk factors, a multicenter and larger sample size study should be conducted.

## Conclusions

Minimally invasive closure of ventricular septal defect has excellent results, no mortality and low incidence of serious adverse events after surgery. Our research shows that the size of the defect is related to the long-term hospitalization after the operation, and the size of the occluder is related to the residual shunt in the early postoperative period. No risk factors related to the occurrence of serious adverse events after the operation were found.

## Data Availability

The datasets used and analyzed during this study are available from the corresponding author on reasonable request.

## Abbreviations

VSD: Ventricular septal defect
TTE: Transesophageal echocardiography
cAVB: Complete atrioventricular block
